# Circular from the War Department

**Published:** 1901-04

**Authors:** John S. Marshall

**Affiliations:** President Examining Board of Dental Surgeons, United States Army; War Department, Surgeon-General’s Office, Washington


### Domestic Correspondence.

### CIRCULAR FROM THE WAR DEPARTMENT.

War Department, Surgeon-General's Office,
Washington, March 16, 1901.

To the Editor:

Sir,—By permission of the Surgeon-General, I have the honor to inform you that the following-named gentlemen have successfully passed the examination before this board, and have received their appointments as Contract Dental Surgeons, United States Army:

Siebert Davis Boak, graduate of National University, Dental Department, Washington, D. C., of Martinsburg, W. Va.

Edward Clarence Lauderdale, graduate of University of Buffalo, Dental Department, Buffalo, N. Y., of Naples, N. Y.

These gentlemen have been ordered to report for duty at San Francisco, Cal., April 15, for service in the Philippines.

At the present writing there have been fourteen gentlemen ordered before this board by the Surgeon-General for examination. Only two, as you see by the above report, have successfully passed the examination. The board has been disappointed in the professional qualifications of most of the young men who have presented themselves. The examination does not cover any subjects which have not been taught in our best dental schools, and the board believes that the questions submitted in the examinations have been of a practical nature, and eminently fair. It is to be hoped, therefore, that our dental schools will not recommend any young men to come before this board who are not thoroughly well qualified, theoretically and practically, in all of the branches comprising the curriculum of our best dental schools.

It will be the pleasure of the Examining Board of Dental Surgeons to keep the profession posted as to its work through the various dental journals.

Kindly acknowledge receipt, and oblige,

Very respectfully,

John S. Marshall,

*President Examining Board of Dental Surgeons, United States Army.*

